# LINC00963-FOSB-mediated transcription activation of UBE3C enhances radioresistance of breast cancer cells by inducing ubiquitination-dependent protein degradation of TP73

**DOI:** 10.1186/s12967-023-04153-z

**Published:** 2023-05-12

**Authors:** Yansu Wang, Ming Liu, Xiaoqian Liu, Xianling Guo

**Affiliations:** 1 Department of Radiotherapy, Shanghai Tenth People’s Hospital, Tongji University, Shanghai, 200072 People’s Republic of China; 2 Department of Oncology, Dermatology Hospital, Tongji University, Shanghai, 200072 People’s Republic of China; 3 Department of Oncology, The Second Affiliated Hospital of Anhui Medical University, Hefei, 230601 Anhui People’s Republic of China; 4 Department of Radiotherapy, Xuzhou Municipal Hospital affiliated of Xuzhou Medical University, 269 Daxue Road, Tongshan District, Xuzhou, 221002 Jiangsu People’s Republic of China; 5 Tongji University Cancer Center, Shanghai, 200072 People’s Republic of China

**Keywords:** Breast cancer, Radioresistance, FOSB, UBE3C, TP73, Ubiquitination

## Abstract

**Background:**

The ubiquitin protein ligase E3C (UBE3C) has been reported to play an oncogenic role in breast cancer (BRCA). This work further investigates the effect of UBE3C on the radioresistance of BRCA cells.

**Methods:**

Molecules linking to radioresistance in BRCA were identified by analyzing two GEO datasets, GSE31863 and GSE101920. UBE3C overexpression or knockdown was induced in parental or radioresistant BRCA cells, followed by irradiation treatment. The malignant properties of cells in vitro, and the growth and metastatic activity of cells in nude mice, were analyzed. Downstream target proteins, as well as upstream transcriptional regulators of UBE3C, were predicted by bioinformatics tools. Molecular interactions were confirmed by immunoprecipitation and immunofluorescence assays. Furthermore, artificial alterations of TP73 and FOSB were induced in the BRCA cells for functional rescue assays.

**Results:**

According to bioinformatics analyses, UBE3C expression was linked to radioresistance in BRCA. UBE3C knockdown in radioresistant BRCA cells reduced while its overexpression in parental BRCA cells increased the radioresistance of cells in vitro and in vivo. UBE3C, which induced ubiquitination-dependent protein degradation of TP73, was transcriptionally activated by FOSB. The radioresistance of cancer cells was blocked by TP73 overexpression or FOSB knockdown. Additionally, LINC00963 was found to be responsible for the recruitment of FOSB to the UBE3C promoter for transcription activation.

**Conclusion:**

This work demonstrates that LINC00963 induces nuclear translocation of FOSB and the consequent transcription activation of UBE3C, which enhances radioresistance of BRCA cells by inducing ubiquitination-dependent protein degradation of TP73.

**Graphical Abstract:**

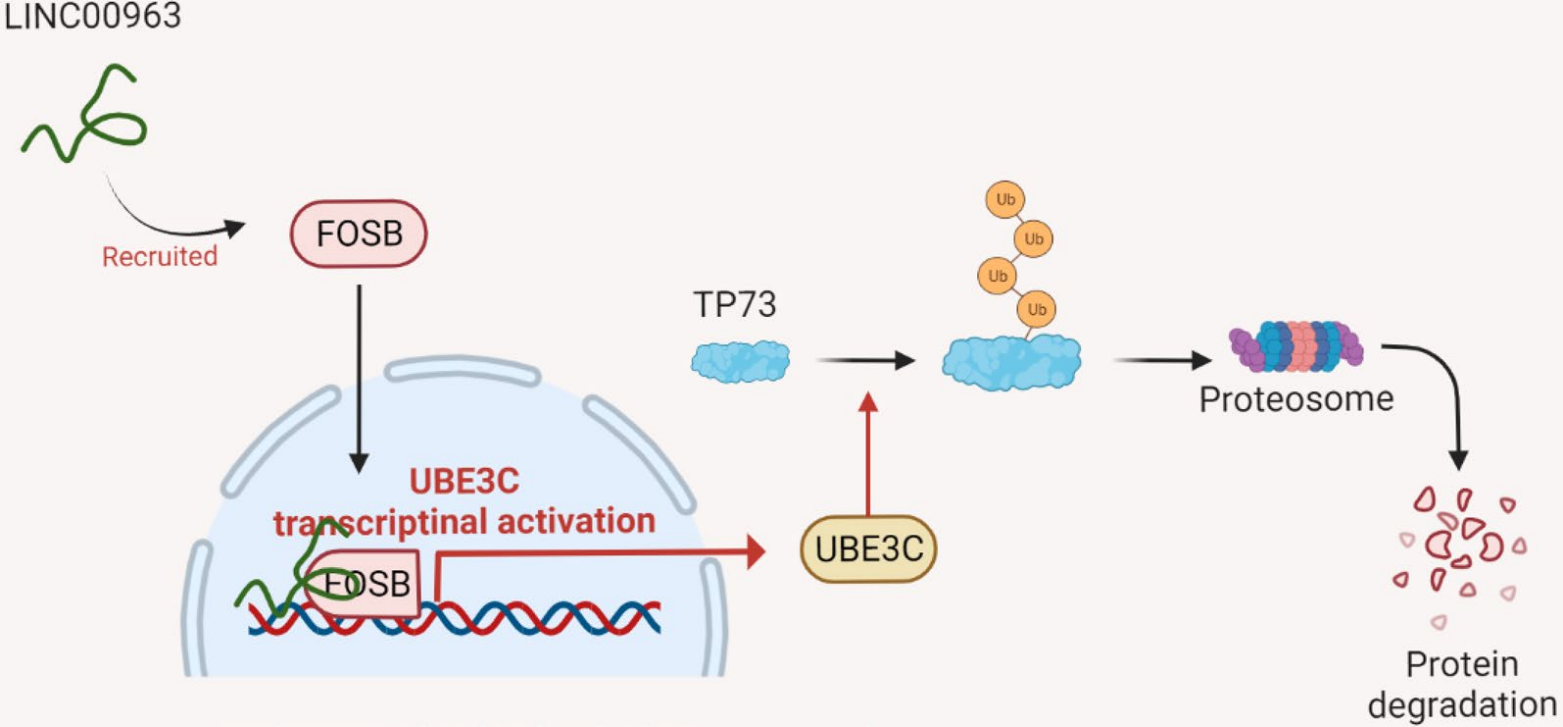

## Introduction

Female breast cancer (BRCA) is ranked as the most common type and the fifth leading cause of cancer-related deaths in 2020 [1]. The mortality rate of BRCA has witnessed a stable decline during the past years, and a timely diagnosis is critical for a better prognosis of patients [2]. The therapeutic strategy for BRCA largely depend on the molecular subtypes, which are generally classified into four classes: luminal A, luminal B, HER-2-positive, and triple-negative [3]. Current standard treatments for BRCA include surgical resection, chemotherapy, radiation therapy, endocrine therapy, and targeted therapy [4]. Radiotherapy is an important option for BRCA, especially for the highly malignant and advanced cancers [5–7]. This sort of therapy uses ionizing radiation (such as X-rays and γ-rays) and has substantially improved the prognosis and survival of BRCA patients after mastectomy [8]. However, the adaptive radioresistance that promotes metastatic and recurrent disease remains a major problem [9].

By analyzing GEO datasets (https://www.ncbi.nlm.nih.gov/gds/) GSE31863 and GSE101920, we obtained ubiquitin protein ligase E3C (UBE3C) as a candidate upregulated in BRCA tissues after radiotherapy. UBE3C (also known as HECTH2) is an E3 ligase, and mutations in the HECT domain of UBE3C can lead to pathophysiological states, such as neurological impairments and cancer in humans [10–12]. In BRCA, UBE3C has been found to activate proliferation, migration, and invasion of cancer cells in vitro by triggering the nuclear translocation of β-catenin, a master factor associated with tumor development [13]. However, there have been no reports on its role in radioresistance to date. E3 ubiquitin ligases primarily induce covalent binding of ubiquitin to target proteins [14, 15]. Proteins ubiquitination generally modulates protein degradation and relocation, which represents a critical post-translational modification that exerts multifaceted functions in cancer-related pathways [16]. In this study, our subsequent bioinformatics analyses predicted tumor protein p73 (TP73) as a potential downstream target of UBE3C, while FosB proto-oncogene (FOSB) was predicted as a potential upstream transcription factor of UBE3C. The TP73 gene encodes protein 73 (p73), a member of the p53 tumor suppressor protein family [17]. TP73 translates into intricate number of isoforms with opposite functions: TAp73 and ΔEx2p73, ΔEx2/3p73, ΔNp73 and ΔN′p73 ([18]. Similar to TP53, TAp73 is a crucial regulator in the cell response to a variety of stress, including DNA damage [19]. Mutation of TP53, as a key tumor-suppressor, has been linked to increased radioresistance of tumor cells [20, 21]. However, the exact role of TP73 in radioresponse in human cancers is not well understood, even though its overexpression has been linked to reduced radioresistance in colorectal cancer [22]. As for FOSB, it has been documented as a fundamental factor supporting migration and invasion of tumor cells [23]. However, its role in radioresistance remains untouched either. In our previous report, we identified that long non-coding RNA (lncRNA) LINC00963 plays significant roles in the radioresistance in BRCA [24]. Intriguingly, we obtained from CatRapidomics (http://service.tartaglialab.com/page/catrapid_omics_group) that LINC00963 has a predicted binding relationship with FOSB. Therefore, we conjectured that LINC00963 possibly affects UBE3C expression by interacting FOSB and therefore affect the protein stability of TP73 to influence radioresistance in BRCA. Collectively, this study aims to clarify the biological functions of UBE3C and its potential interacting molecules including FOSB and TP73 in radioresistance of BRCA cells both in vitro and in vivo.

## Materials and methods

### Bioinformatics

Two datasets GSE31863 and GSE101920 concerning molecular signature of genes related to radiosensitivity in BRCA were downloaded from the GEO database (https://www.ncbi.nlm.nih.gov/gds). The Limma R Package was used to normalize and adjust the the datasets. Differentially expressed genes (DEGs) were screened by LogFC > 2.0 and adj. *p* value < 0.05. Expression of the top 10 DEGs in BRCA, and the correlation between UB3E3C expression with BRCA patient’s clinical stages and prognosis were further queried in the Gene Expression Profiling Interactive Analysis (GEPIA; http://gepia2.cancer-pku.cn/) system, a web-based tool that allows users to analyze RNA sequencing data of tumors and normal samples from The Cancer Genome Atlas (TCGA; https://www.cancer.gov/research/areas/genomic) project. Correlation of UBE3C with the radiosensitivity in TCGA-BRCA was analyzed in the UCSCXena database (https://xenabrowser.net/). Possible proteins that can bind with UBE3C were predicted in Ubibrowser (http://ubibrowser.bio-it.cn/ubibrowser_v3/). The promoter sequence of UBE3C was predicted from UCSCbrowser (http://genome.ucsc.edu/), and then the candidate transcription factors that can bind with UBE3C promoter were predicted from JASPAR (https://jaspar.genereg.net/). The ChIP-seq data of the candidate transcription factors in the breast cancer cell line MCF-7 were downloaded from ENCODE (https://www.encodeproject.org/), and the binding peaks of FOSB (ENCSR569XNP), ZNF302 (ENCFF037UPH), SP1 (ENCFF072FPF), ELK1 (ENCFF123KER) and FOXF2 (ENCFF008ABX) with the UBE3C promoter were analyzed. The genomic data were visualized and analyzed by the UCSC browser (https://genome.ucsc.edu/). Additionally, RNA molecules that can bind with UBE3C and proteins that can bind with LINC00963 were predicted in CatRapidomics.

### Cells

MDA-MB-23 L (CRM-HTB-26), SK-BR-3 (HTB-30) and HEK-293T (ACS-4500) cell lines were procured from ATCC. The MDA-MB-23 L and SK-BR-3 cell lines were cultured in RPMI-1640 containing 10% fetal bovine saline (FBS) and 1% penicillin/streptomycin. All cells were cultured in six-well plates containing 5 µL short hairpin (sh) RNA and 5 µL Lipofectamine 2000 (Thermo Fisher Scientific, Rockford, IL, USA). The shRNA carrier Ribo™ h-UBE3C Smart Silencer was provided by RiboBio Co., Ltd., (Guangzhou, Guangdong, China). ShRNA targeting UBE3C, TP73 and FOSB were provided by Origene, and the sequence information is given in Table 1.

**Table 1:** shRNA sequences

Symbol	Ds Oligos
UBE3C-#1	5'-CACCGCATTTGATCGCTGTGCTACCCGAAGGTAGCACAGCGATCAAATGC-3'
UBE3C-#2	5'-CACCGGATGGATCTGAGAGACTTACCGAAGTAAGTCTCTCAGATCCATCC-3'
TP73	5'-CACCAGCCAGTTGACAGAACTAAGGCGAACCTTAGTTCTGTCAACTGGC-3'

Cells in the six-well plates were also incubated with 1 µg gene overexpression vector of UBE3C, TP73, or FOSB, or with 3 µL Polyjet (SignaGen Laboratories) for 24 h. The gene overexpression vectors were provided by GeneChem Co., Ltd. (Shanghai, China). In the present paper, gene silencing was designated as knockdown (kd) while gene overexpression as knockin (ki). Scramble shRNA was set as the control (Con) for shRNAs while Empty vector (Vec) was set for the control for gene overexpression vectors.

### Reverse transcription quantitative polymerase chain reaction (RT-qPCR)

Total RNA from the cultured cells or tissues was isolated using the TRIzol reagent (Thermo Fisher Scientific). Reverse transcription of RNA to cDNA was conducted using a commercially acquired cDNA synthesis kit (Thermo Fisher Scientific). Thereafter, qPCR analysis was performed using the SYBR Green Master Mix (Thermo Fisher Scientific). Relative gene expression was evaluated using the 2^−ΔΔCt^ method with actin beta (ACTB) mRNA as the endogenous control. The primer information is provided in Table 2.

**Table 2:** Primers

Gene	Fvd	Rvs
UBE3C	ATGAACCTGCTGAAGCTCCC	CTGACGAAGGAAGCACTGGT
FOSB	TTTTCTCCTCCGCCTGTGTC	TCACACTCTCACACTCGCAC
TP73	GGGAGGGACTTCAACGAAGG	ATGGTGGTGAATTCCGTCCC
LINC00963	CTGTGTTACCCTGGCTGGAG	AAATGACTCAGGCTGGGCTC
γH2AX	ACGACGAGGAGCTCAACAAG	CGGGCCCTCTTAGTACTCCT
GAPDH	GATTTGGTCGTATTGGGCGC	TTCCCGTTCTCAGCCTTGAC

### Western blot (WB) analysis

Total protein was obtained by the RIPA lysis buffer (Beyotime Biotechnology Co., Ltd., Shanghai, China). According to the bicinchoninic acid analysis, the protein concentration was 1.5–2 µg/µL. After that, equal amounts of protein samples (50 µg) were separated by sodium dodecyl sulfate-polyacrylamide gel electrophoresis and transferred onto polyvinylidene fluoride membranes. The membranes were blocked with 5% non-fat milk for 15 min and covered with the diluted primary antibodies including ACTB (1:2,000, ab8226, Abcam Inc., Cambridge, MA, USA), FOSB (1:1,000, MA5-15056, Thermo Fisher Scientific), TP73 (1:1000, ab202474, Abcam), UBE3C (1:1,000, PA5-110540, Thermo Fisher Scientific), and γH2AX (1:2,000, ab81299, Abcam) overnight at 4 ℃. Later, the membranes were further incubated with secondary antibody (1:5,000, GTX213110-01, GeneTex Inc.) at room temperature for 1 h. The protein bands were developed using the enhanced chemiluminescence kit (Thermo Fisher Scientific), and the protein level relative to ACTB was analyzed by Image J.

### Colony formation assay

Exponentially growing parental or radioresistant SK-BR-3 and MDA-MB-231 cells were cultured in six-well plates at a density of 300 cells per well at 37 °C with 5% CO_2_. After two weeks, when visible cell colonies were observed, the cells were rinsed with PBS, and the cell colonies were fixed with formaldehyde for 10 min and stained with Giemsa working solution for 15 min. The cell colonies were counted under the microscope.

### CellTiterGlo (CTG) assay

Cell viability after 8 Gy irradiation was assessed using the CellTiter-Glo®2.0 kit (Promega; G9242) according to the manufacturer’s protocol. Briefly, cells were seeded at 5000 cells/well in 96-well plates (Corning Glass Works, Corning, NY, USA) and incubated overnight. Afterward, the cells were exposed to 8 Gy irradiation for 2 h, and then the CTG reagent was added to the wells. The luminescence signal was read by a plate reader (BioTek Instruments Inc., Winooski, VT, USA). Relative survival was normalized to the DMSO treatment group.

### Flow cytometry

Exponentially growing cells were seeded into culture flasks at 2.5 × 10^6^ cells/mL. After 24 h, the cells were cultured in RPMI-1640 containing 2% FBS for 24 h. Thereafter, the cells were collected, stained following the instructions of AnnexinV FITC/PI kit (Beyotime), and then the apoptosis of cells in each group was detected by flow cytometry. The Annexin-V^+^/PI^−^ cells were defined as early apoptotic cells, and the Annexin-V^+^/PI^+^ cells were defined as late apoptotic cells.

### Terminal deoxynucleotidyl transferase (TdT)-mediated dUTP nick end labeling (TUNEL)

TUNEL assay was performed using an in-situ cell death detection kit (Sigma-Aldrich Chemical Company, Merck KGaA, Darmstadt, German). The tissues were prepared as 4-µm thick sections. The sections were deparaffinized and rehydrated using standard protocols, followed by treatment with 50 µL Protein K working solution (1×) at 37 °C for 25 min. Later, the sections were warm-incubated with the TUNEL reaction mixture (1:9) containing TdT and dUTP at 37 °C for 2 h in a humidified environment and then counter-stained with DAPI and analyzed under a fluorescence microscope. The apoptosis index (AI) was analyzed by Image-ProPlus6.0 software, and the calculation formula was as follows: ${\text{ AI = (the number of TUNEL - positive nuclei in one area)/(the number of total nuclei in the same area) }}$

### Immunofluorescence staining and in-situ hybridization

To visualize the sub-cellular location of LINC00963 and proteins, BRCA cells were fixed, permeabilized, and prehybridized. After that, we performed the hybridization with Cy-3-conjugated LINC00963 probes in the dark at 37 °C overnight. Thereafter, the cells were rinsed at 42 °C in SSC buffer and blocked with 5% bovine serum albumin (BSA). Subsequently, the cells with reacted with the primary antibody at room temperature for 1 h and then with secondary antibody, followed by and DAPI staining and microscopy observation.

### Xenograft mouse models

The animal study protocol was approved by the hospital institutional review board of Shanghai Tenth People’s Hospital, Tongji University. Male nude mice (BALB/c, 8 weeks old) were procured from SJA Laboratory Animal Co., Ltd. (Hunan, China). The parental or radioresistant BRCA cells with corresponding stable transfections (using lentivirus vectors) were injected into the nude mice subcutaneously. From day 15, the mice were exposed to 16 Gy irradiation once every three days. The length (L) and width (W) of the tumors were measured to calculate the volume (V) as follows: V (mm^3^) = L × W^2^ × 0.5. At the end point of experiments (day 35), the mice were euthanized by overdosed pentobarbital Na and the tumors were collected and weighed.

Additionally, metastasis of BRCA cells was analyzed in vivo. The luciferase-containing parental or radioresistant BRCA cells (2 × 10^6^ cells) were injected into the nude mice via intracardiac injection. The fluorescence images were obtained by the imaging system (Ex/Em = 710/790 nm) to analyze the fluorescence intensity. After 35 d, the animals were euthanized, and the lung tissues were collected for hematoxylin and eosin staining.

### Immunohistochemistry (IHC)

The tumor tissue sections were embedded and prepared as 5-µm sections. The sections were deparaffined, rehydrated, treated with H_2_O_2_ and Tris/EDTA buffer (pH 9.0), and then blocked with 5% BSA for 20 min. After that, the sections were incubated with the antibodies including KI67 (ab16667, Abcam), PCNA (ab18197, Abcam) and γH2AX (ab81299, Abcam) at 4 ℃ overnight, and then with the secondary antibody at room temperature for 2 h. After color development by DAB and nuclei counter-staining with hematoxylin, the sections were sealed for microscopy observation to evaluate the positive staining (brownish staining).

### Co-immunoprecipitation (Co-IP)

For Co-IP assay, magnetic beads were pre-incubated with the antibody of P73 (1:100, ab202474, Abcam). After that, the cells were lysed in with Co-IP buffer and centrifuged. The supernatant was collected and incubated with the beads overnight at 4 ℃. Finally, the beads were collected and the protein levels in the immunoprecipitates were examined by WB analysis using anti-UB antibody (1:1000, ab140601, Abcam).

### Ubiquitination examination in vitro

In brief, 1 µg GST-TP73, 500 ng GST-UBE3C, 10 µg FLAG-tagged ubiquitin, 200 ng E1 and E2 ubiquitination ligases were added to the reaction system, followed by the addition of ubiquitination buffer (25 mM HEPES, pH 7.4; 3 mM MgCl_2_; 10 mM NaCl; 0.05% Triton-X-100; 0.5 mM DTT, 3 mM Mg-ATP; 1% protease inhibitor cocktail) till a final concentration of 50 µL. After reaction at 37 °C for 1 h, the product was run on SDS-PAGE for WB analysis.

### Chromatin immunoprecipitation (ChIP)-qPCR

The ChIP assay was performed according to the instructions of the ChIP kit (Cell Signaling Technology (CST), Beverly, MA, USA). In brief, cells were crosslinked in formaldehyde, and the reaction was terminated by glycine. Later, the cell lysate was collected and ultrasonicated for DNA truncation. The supernatant was collected and reacted with anti-FOSB (MA5-15056, Thermo Fisher Scientific) or normal rabbit IgG at 4 °C for 1 h, and then incubated with protein A/G magnetic beads overnight. Thereafter, the protein A beads was collected and washed, and the DNA was eluted and purified, and the UBE3C promoter fragment was quantified by qPCR analysis.

### Luciferase reporter gene assay

The UBE3C promoter sequence was inserted into the pGL3-Basic vector (Promega Corporation, Madison, WI, USA) to construct luciferase reporter vector. The empty vector pGL3-enhancer plasmid was used as the negative control, and the pGL3-control expressing the firefly luciferase was selected as the positive control. The recombinant vectors pGL3-UBE3C-promoter and pGL3-UBE3C-promoter-Nluc-E were used as the experimental groups. The pRL-SV40 plasmid containing renal luciferase was used as an internal control. Overexpression vectors of FOSB or LINC00963 was transfected into cells to examine the alteration in luciferase activity.

### Biotin-labeled RNA pull-down assay

Biotinylated LINC00963 was prepared using the MEGAscript™ T7 Transcription Kit (Invitrogen) and Pierce RNA 3′ End Desthiobiotinylation Kit (Thermo Fisher Scientific) following the manufacturer’s protocol. The RNA-protein pull-down was then performed using the Pierce Magnetic RNA-Protein Pulldown Kit (Thermo Fisher Scientific). Briefly, biotinylated RNA was captured with streptavidin-coated magnetic beads and incubated with whole-cell lysates of cells at 4 °C for 6 h. The recovered eluates were separated by SDS-PAGE for subsequent analysis. 

### RNA immunoprecipitation (RIP)

Cells were lysed in RIPA to collect protein sample. The protein sample was reacted with the primary antibodies at 4 ℃ overnight. The antibody-conjugated sample was reacted with protein A Sepharose (Sigma-Aldrich) at 4 ℃ for 2 h. After that, the sample was washed and incubated with proteinase K for 1.5 h. The RNA in the sample was extracted by TRIzol reagent and examined by RT-qPCR.

### Statistical analysis

SPSS 21.0 (IBM Corp. Armonk, NY, USA) was used for data processing. Normal distribution of measurement data was examined by Kolmogorov-Smirnov test, and the data are presented as the mean ± standard deviation. Differences between groups were compared by the *t* test, or by the 1 or 2-way ANOVA when multiple groups are included. *p* < 0.05 was deemed to represent significant difference.

## Results

### UBE3C is upregulated in BRCA samples after radiotherapy

**Fig. 1:**
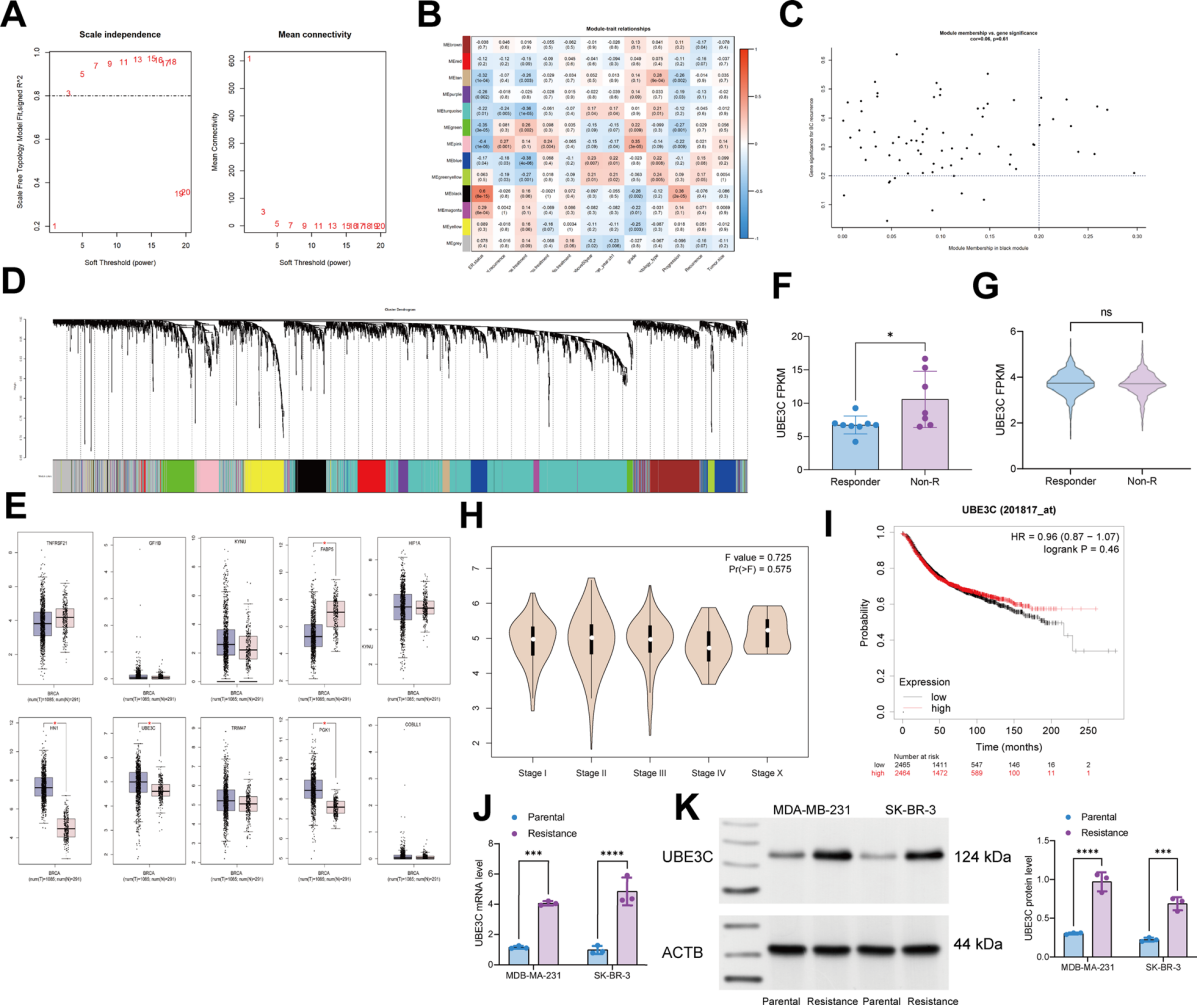
UBE3C is upregulated in BRCA samples after radiotherapy. **A** analysis of network topology for various soft-thresholds (power) in the dataset; **B** heatmap for the correlation between the WGCNA gene clustering module and clinical traits; **C** correlation of black modules with clinical traits; **D** modules for each TCGA-WGCNA gene (each branch represents one gene); **E** expression of the top 10 DEG (TNFRSF21, GFI1B, KYNU, FABP5, HIF1A, HN1, UBE3C, TRIM47, PGK1, COBLL1) in TCGA-BRCA; **F** UBE3C expression in the GSE101920 dataset; **G** UBE3C expression in patients with or without response to radiotherapy in TCGA-BRCA; **H**, **I**, correlations between UBE3C expression and the clinical stage (**H**) and survival (**I**) of patients in TCGA-BRCA; **J**, **K** RT-qPCR and WB analyses for mRNA (**J**) and protein (**K**) levels of UBE3C in Pa and R MDA-MB-231 and SK-BR-3 cells. Data are expressed as the mean ± SD. Three repetitions were performed. Differences were analyzed by two-way ANOVA. **p < 0.01, ***p < 0.001

We downloaded the GEO dataset GSE31863 which contains BRCA tissues that either respond well (n = 29) or not (n = 114) to radiotherapy. We established a co-expression network by setting the soft threshold at 5 (Fig. 1A) and a cutting height at 120 (Fig. 1B). We analyzed the associations between ME values and sample traits to determine the potential correlations between these modules and clinical characteristics, and it was found that the black module was most closely linked to the progression (Fig. 1C–D). We identified 10 key genes with correlation coefficients over 0.3 among the candidate key genes in response to radiotherapy, namely TNFRSF21, GFI1B, KYNU, FABP5, HIF1A, HN1, UBE3C, TRIM47, PGK1, COBLL1. Among them, UBE3C showed the greatest degree of differential expression between normal and BRCA samples in TCGA-BRCA (Fig. 1E). Furthermore, to validate the correlation between UBE3C and radioresistance in breast cancer, we downloaded the radiotherapy transcriptome microarray dataset (GSE101920) of BRCA patients from the GEO database, in which we found that UBE3C was also significantly increased after radiation treatment (Fig. 1F). By analyzing the TCGA-BRCA data through the UCSCXena tool, we found that the expression UBE3C was elevated in poor-responding patients, though there is no statistical significance (Fig. 1G). Additionally, according to TCGA-BRCA data, UBE3C was strongly linked to advanced clinical stage and poor prognosis of patients (Fig. 1H, I). Therefore, we believed that UBE3C has specific links to radioresistance of BRCA patients. To validate this, we examined the mRNA and protein expression levels of UBE3C in parental (Pa) and radioresistant (R) BRCA cells (MDA-MB-231-Pa, SK-BR-3-Pa, MDA-MB-231-R and SK-BR-3-R). Indeed, the UBE3C expression was significantly elevated in the radioresistant cell lines (Fig. 1J, K).

### UBE3C knockdown enhances radiosensitivity of BRCA cells

**Fig. 2:**
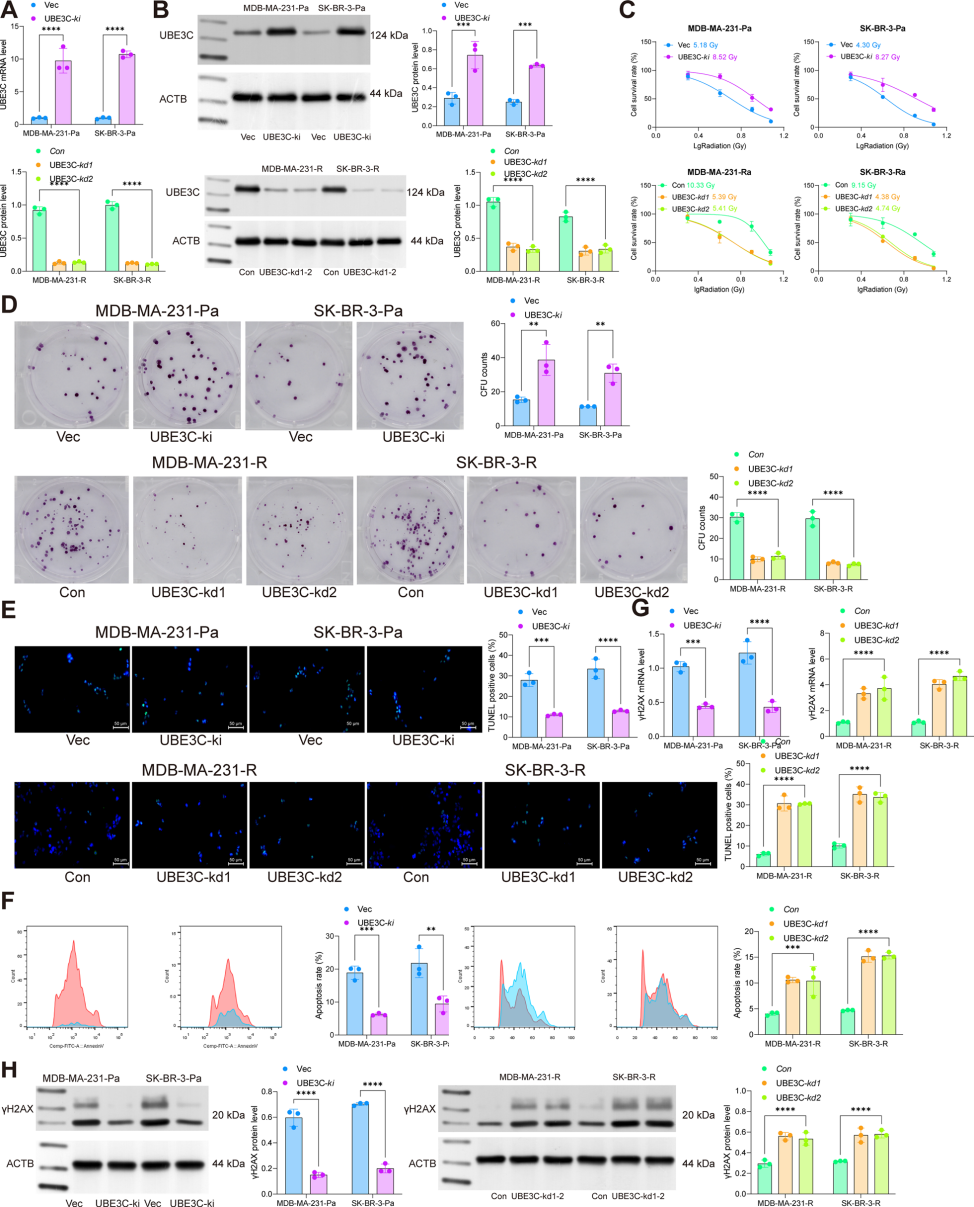
UBE3C knockdown enhances radiosensitivity of BRCA cells. A, RT-qPCR and WB analyses for mRNA (**A**) and protein (**B**) levels of UBE3C in MDA-MB-231 and SK-BR-3 cells (R and Pa) after sh-UBE3C or oe-UBE3C transfection; **C** sensitivity of MDA-MB-231 and SK-BR-3 cells (R and Pa) to different doses of irradiation ( 0, 2, 4, 8, and 12 Gy) analyzed by the CTG kit; **D**, number of colonies formed by R or Pa BRCA cells under 8 Gy irradiation determined by colony formation assay; **E** apoptosis of cells analyzed by flow cytometry; **F**, number of apoptotic bodies formed by cells analyzed by TUNEL assay; **G**, **H** RT-qPCR and WB assays for the mRNA (**G**) and protein (**H**) expression of γH2AX in cells. Data are expressed as the mean ± SD. Three repetitions were performed. Differences were analyzed by two-way ANOVA. **p < 0.01, ***p < 0.001

To figure out the effect of UBE3C on radioresistance in BRCA, we induced UBE3C knockdown (UBE3C-kd) in MDA-MB-231-R and SK-BR-3-R cells using shRNA while UBE3C knock-in (UB3EC-ki) in MDA-MB-231-Pa and SK-BR-3-Pa using overexpression vectors. The successful knockdown or overexpression of UBE3C in cells was confirmed by RT-qPCR and WB assays (Fig. 2A, B). The cells were then exposed to 0, 2, 4, 8 or 12 Gy irradiation. It was clearly shown that the UBE3C knockdown reduced whereas the its overexpression promoted the radioresistance in the R or Pa BRCA cells, respectively (Fig. 2C). Later, the cells were treated with 8 Gy irradiation. It was observed that the UBE3C knockdown in R cells decreased the number of cell colonies (Fig. 2D) and induced cell apoptosis (Fig. 2E). Moreover, the TUNEL assay revealed that the UBE3C knockdown induced DNA break and the formation of apoptotic bodies (Fig. 2F). RT-qPCR and WB assays further identified elevated levels of γH2AX, a DNA damage marker, in the MDA-MB-231-R and SK-BR-3-R cells under 8 Gy irradiation exposure (Fig. 2G, H). On the other hand, in MDA-MB-231-Pa and SK-BR-3-Pa cells with artificial UBE3C knock-in, the cells showed significantly increased radioresistance, increased colony formation ability, reduced apoptosis, and alleviated DNA damage under 4 Gy irradiation exposure (Fig. 2C–H).

### Knockdown of UBE3C reduces radioresistance of BRCA cells in vivo

**Fig. 3:**
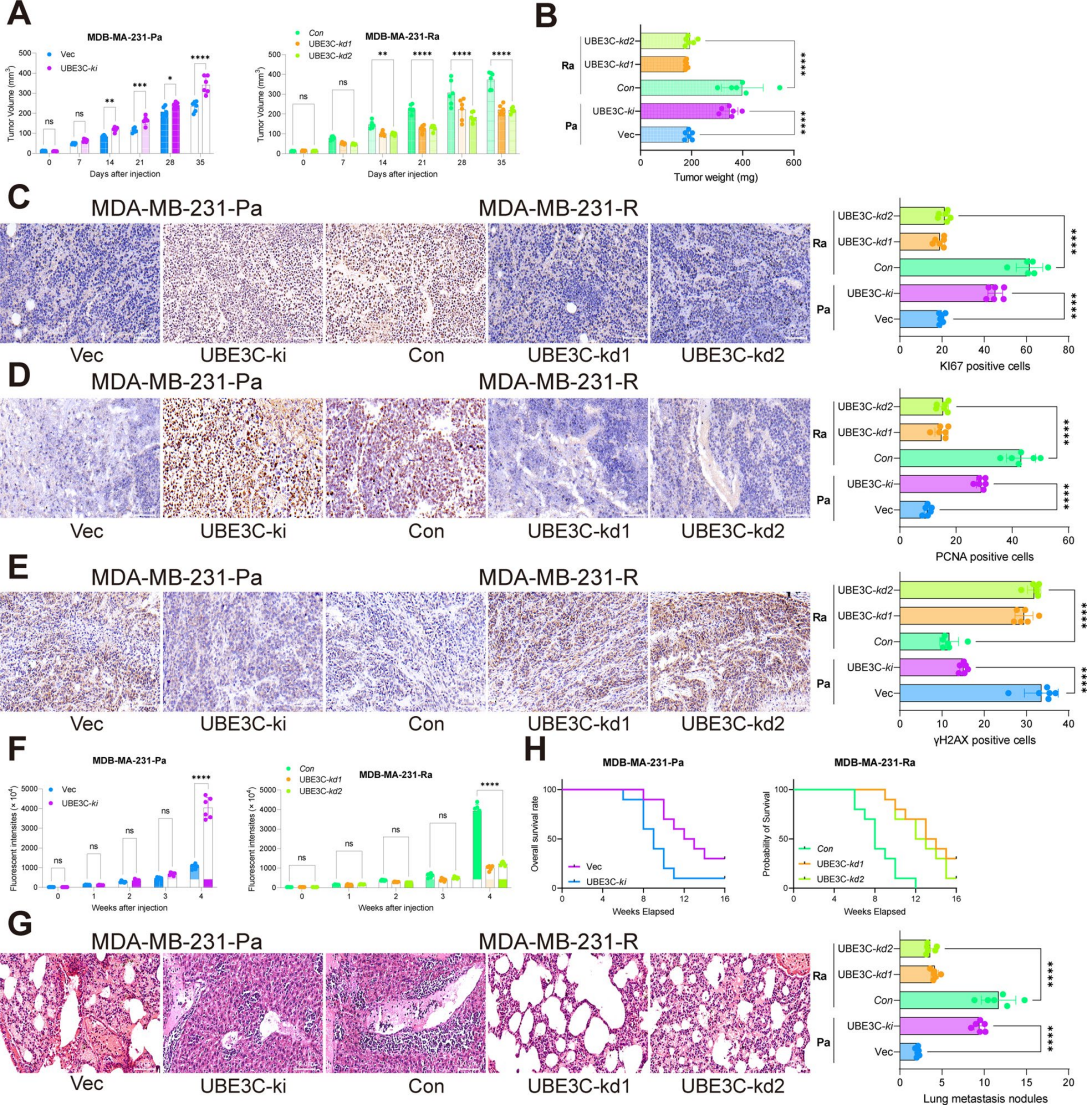
Knockdown of UBE3C reduces radioresistance of BRCA cells in vivo. **A**, **B** R cells transfected with sh-UBE3C and Pa cells transfected with oe-UBE3C were injected into mice subcutaneously, followed by 16 Gy irradiation once every 3 d. Then, the volume of tissues was measured every 5 d (**A**); the tumors were collected and weighed on day 35 after animal euthanasia (**B**); **C–E** staining intensity of KI67 (**C**) PCNA (**D**) and γH2AX (**E**) in xenograft tumor tissues analyzed by IHC; **F** R and Pa cells were further transfected with luciferase and injected into mice intracardiacally, followed by 16 Gy irradiation once every 3 d as well. Later, the dissemination of tumor cells was analyzed by in-vivo imaging analysis once every 5 d for a total of 30 d; **G** number of metastatic nodules in mouse lung tissues; **H** survival analysis of mice in each group within 16 weeks (Kaplan-Meier analysis). In each group, n = 6 in each group. Data are expressed as the box-and-whisker plots. Differences were analyzed by one- or two-way ANOVA followed by Tukey’s multiple test. **p < 0.01, ***p < 0.001

To further examine the correlation of UBE3C with radioresistance in BRCA, the MDA-MB-231-R cells with stable UBE3C-kd and MDA-MB-231-Pa cells with stable UBE3C-ki were injected subcutaneously into the mice, followed by 16 Gy irradiation once every 3 d. It was found that the parental cells overexpressing UBE3C had a significantly increased growth rate and reduced sensitivity to RT. On the other hand, R cells with UBE3C knockdown showed significantly enhanced radiosensitivity and reduced growth rate in vivo (Fig. 3A, B). The IHC results showed that the staining intensity of KI67, PCNA, and γH2AX in xenograft tumors was reduced with UBE3C downregulation but promoted with UBE3C upregulation (Fig. 3C–E).

The parental and R cells, with corresponding UBE3C-kd or UBE3C-ki induction, were further transfected with luciferase and injected into mice intracardiacally to analyze tumor cell metastasis. The mice were given 16 Gy irradiation once every 3 d as well. Similarly, the luciferase activity of Pa cells rapidly increased from day 5. In contrast, the luciferase activity of R cells was decreased by irradiation (Fig. 3F). The lung tissues were collected for HE staining. It was found that the number of metastatic nodules in the lung tissues was increased upon UBE3C overexpression but decreased upon UBE3C knockdown (Fig. 3G), which showed a positive correlation with the luciferase distribution in vivo. Another 10 mice were collected in each group for survival analysis over a period of 16 weeks, during which they received 16 Gy irradiation once every 3 d. It was observed that the mice with UBE3C overexpression had a shorter survival period, whereas those with UBE3C knockdown showed a better survival rate (Fig. 3H).

### UBE3C binds to TP73 and inhibits the protein stability

**Fig. 4:**
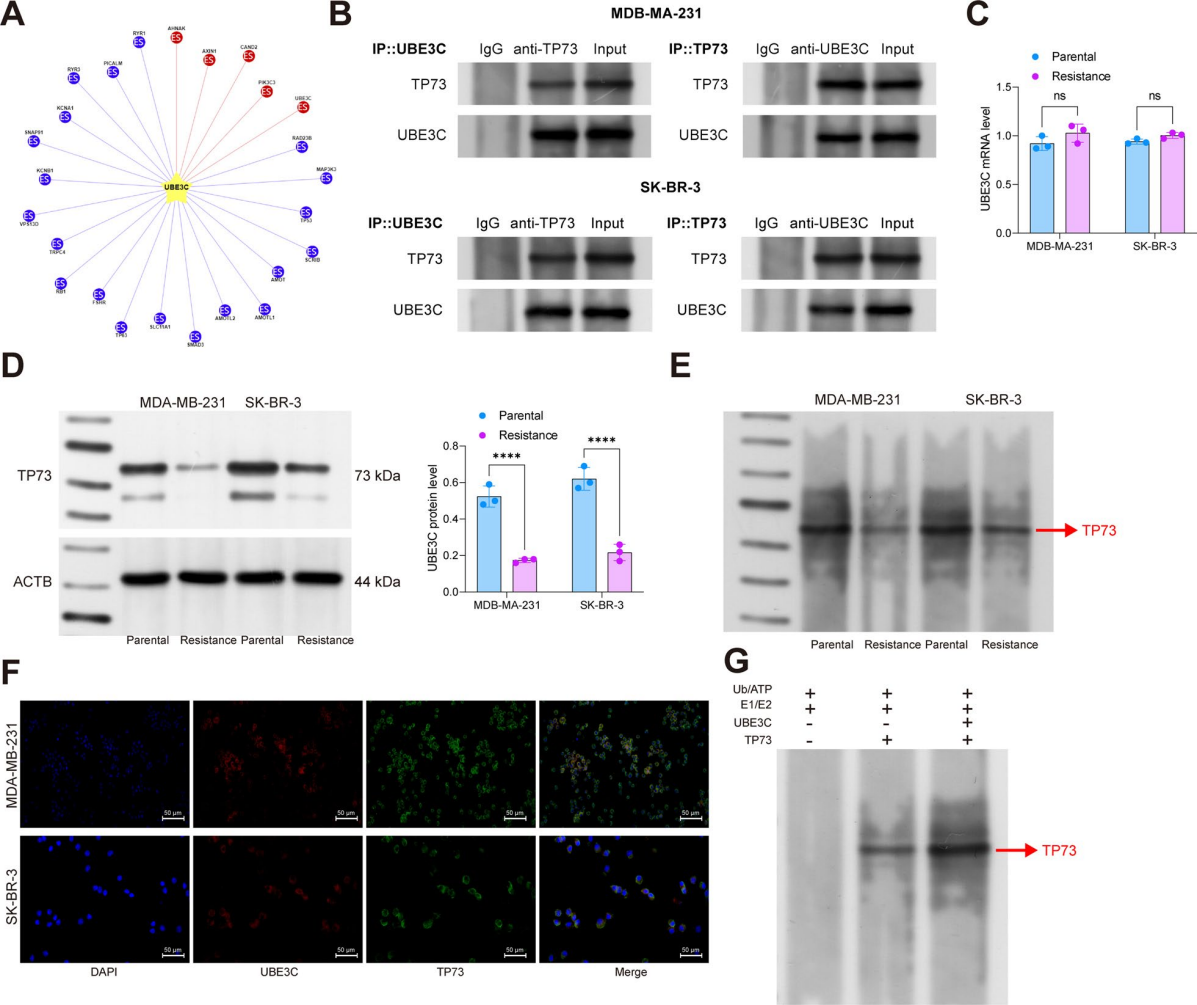
UBE3C binds to TP73 and inhibits the protein stability. **A** proteins that can bind to UBE3C predicted via Ubibrowser; **B** protein level of TP73 in the complexes pulled down by anti-UBE3C examined by WB analysis; **C**, **D** mRNA and protein levels of TP73 in parental and radioresistant MDA-MB-231 and SK-BR-3 cells analyzed by RT-qPCR and WB analyses; **E** ubiquitination level of TP73 in MG132-treated MDA-MB-231 and SK-BR-3 cells; **F**, binding of UBE3C with TP73 in MDA-MB-231 and SK-BR-3 cells determined by double-label immunofluorescence staining; **G** the effect of UBE3C on TP73 ubiquitination modification analyzed by the in vitro ubiquitination assay. Data are expressed as the mean ± SD. Three repetitions were performed. Differences were analyzed by two-way ANOVA (**B–G**). **p < 0.01, ***p < 0.001

As an E3 ubiquitin ligase, UBE3C mainly exerts function by regulating protein stability. Therefore, we explored the possible binding proteins of UBE3C via the Ubibrowser system and obtained that UBE3C can potentially bind to RAD23B, MAP3K3, TP73, SCRIB, and AMOTL2 (Fig. 4A). Therefore, Co-IP assays were performed, which showed that TP73 owned the highest enrichment level in the complex pulled down by anti-UBE3C (Fig. 4B). A previous literature demonstrates that miR-126 promotes Tamoxifen tolerance in BRCA by inhibiting the expression of TP73 [25]. Therefore, we hypothesized that UBE3C possibly contribute to the radioresistance of BRCA cells by promoting the protein instability of TP73. To evaluate this hypothesis, we examined the expression of TP73 in cells. Consistent with the conjecture, the mRNA expression showed little difference between the parental and radioresistant cell lines; however, the protein level of TP73 was significantly reduced in the radioresistant cell lines (Fig. 4C, D). Later, the cells were treated with MG132, a proteasome inhibitor, followed by immunoprecipitation assay using anti-TP73 and immunoblot analysis using anti-UB to examine the TP73 ubiquitination level in parental and radioresistant cell lines, respectively. Of note, the radioresistant cells showed significantly elevated level of ubiquitination (Fig. 4E). The double-label immunofluorescence staining regarding the localization of UBE3C and TP73 proteins showed that the UBE3C (red) and TP73 (green) fluorescence was mainly overlapped (yellow) in cytoplasm (Fig. 4F). Furthermore, the in vitro ubiquitination assay showed that upon UBE3C overexpressing vector transfection, the TP73 showed significantly elevated ubiquitination levels (Fig. 4G). These results preliminarily indicate that aberrant high expression of UBE3C in radioresistant BRCA cells induces ubiquitination and protein degradation of TP73.

### Overexpression of TP73 reduces radioresistance of BRCA cells

**Fig. 5:**
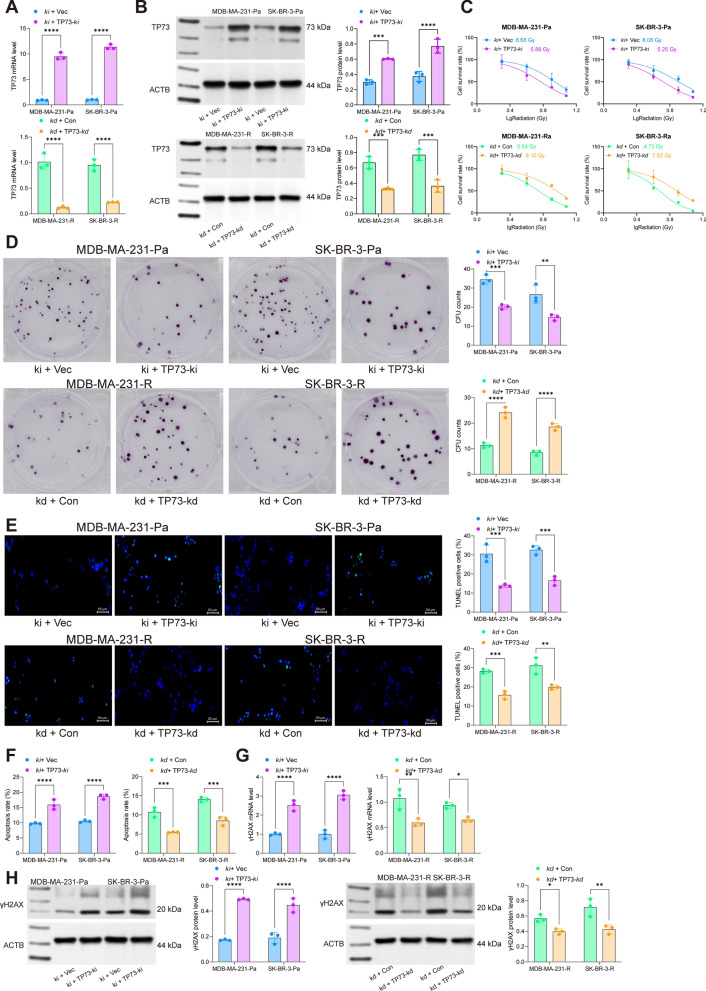
Overexpression of TP73 reduces radioresistance of BRCA cells. A, RT-qPCR and WB analyses for mRNA (**A**) and protein (**B**) levels of TP73 in MDA-MB-231 and SK-BR-3 cells (R and Pa) after sh-TP73 or oe-TP73 transfection; **C** sensitivity of MDA-MB-231 and SK-BR-3 cells (R and Pa) to different doses of irradiation ( 0, 2, 4, 8, and 12 Gy) analyzed by the CTG kit; **D** number of colonies formed by R or Pa BRCA cells under 8 Gy irradiation determined by colony formation assay; **E** apoptosis of cells analyzed by flow cytometry; **F** number of apoptotic bodies formed by cells analyzed by TUNEL assay; **G**, **H** RT-qPCR and WB assays for the mRNA (**G**) and protein (**H**) expression of γH2AX in cells. Data are expressed as the mean ± SD. Three repetitions were performed. Differences were analyzed by two-way ANOVA. **p < 0.01, ***p < 0.001

To unravel the exact function of TP73 in the radiosensitivity of BRCA cells, the MDA-MB-231-R and SK-BR-3-R cells transfected with UBE3C-kd were further administrated with TP73-kd. Correspondingly, the MDA-MB-231-Pa and SK-BR-3-Pa cells were administrated with UBE3C-ki followed by TP73-ki. The successful knockdown or overexpression of TP73 was confirmed by RT-qPCR and WB assays (Fig. 5A, B). Of note, it was found that the radioresistance in parental cells triggered by UBE3C was significantly blocked by additional TP73 knock-in, as manifested by increased cell apoptosis, γH2AX expression, and reduced cell colonies following 8 Gy irradiation (Fig. 5C–H). In contrast, in the radioresistant cells, the radiosensitivity increased by UBE3C-kd was diminished upon further TP73 knockdown (Fig. 5C–H).

### UBE3C is transcriptionally regulated by FOSB

**Fig. 6:**
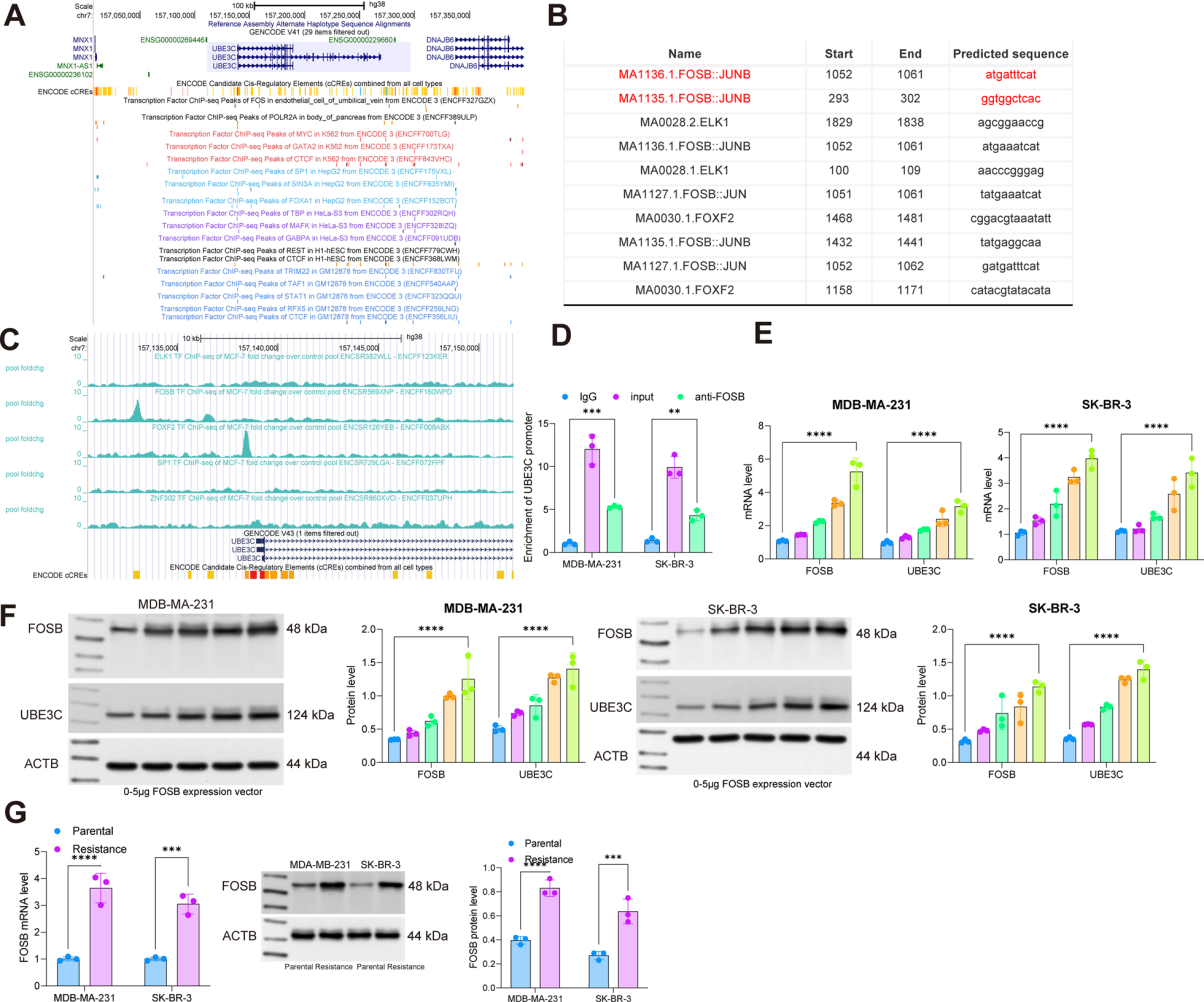
UBE3C is transcriptionally regulated by FOSB. **A** transcription factors that can bind to UBE3C promoter predicted in UCSCbrowser;** B** transcription factors that can bind to UBE3C promoter and the conservative binding sequences obtained from JASPAR; **C** ChIP-seq data of FOSB (ENCSR918GHT), ZNF302 (ENCSR590IHT), SP1 (ENCSR000BPE), ELK1 (ENCSR623KNM) and FOXF2 (ENCSR445FHB) in a lung cancer cell line A549; **D** binding of FOSB and UBE3C promoter examined by ChIP-qPCR; **E**, **F** RT-qPCR and WB assays for the mRNA (**E**) and protein (**F**) expression of FOSB and UBE3C in MDA-MB-231 and SK-BR-3 cells following oe-FOSB transfection. Data are expressed as the mean ± SD. Three repetitions were performed. Differences were analyzed by two-way ANOVA. **p < 0.01, ***p < 0.001

To unravel the upstream mechanism of UBE3C, we obtained the promoter sequence of UBE3C from UCSCbrowser and explored the possible transcription factors that can bind to UBE3C promoter by JASPAR. Five candidate factors, including ZNF302, FOXF2, SP1, FOSB, and ELK1 were predicted to have a putative binding with the UBE3C promoter (Fig. 6A, B). We obtained ChIP-seq data of the candidate transcription factors FOSB (ENCSR569XNP), ZNF302 (ENCFF037UPH), SP1 (ENCFF072FPF), ELK1 (ENCFF123KER), and FOXF2 (ENCFF008ABX) in a breast cancer cell line MCF-7 from ENCODE. The results showed that FOSB had the highest binding peak with the UBE3C promoter (Fig. 6C). To further confirm the binding between FOSB and UBE3C promoter, we performed ChIP assay in the MDA-MB-231 and SK-BR-3 cells and identified abundant UBE3C promoter fragments in the complexes reacted by anti-FOSB (Fig. 6D). Additionally, we found that the artificial overexpression FOSB in the MDA-MB-231 and SK-BR-3 cells elevated UBE3C expression in a dose-dependent manner (Fig. 6E, F). Moreover, the RT-qPCR and WB assays identified elevated mRNA and protein levels of FOSB in the radioresistant cells compared to the parental cells (Fig. 6G, H), which might be the cause for the UBE3C upregulation.

### FOSB knockdown suppresses radioresistance of BRCA cells

**Fig. 7:**
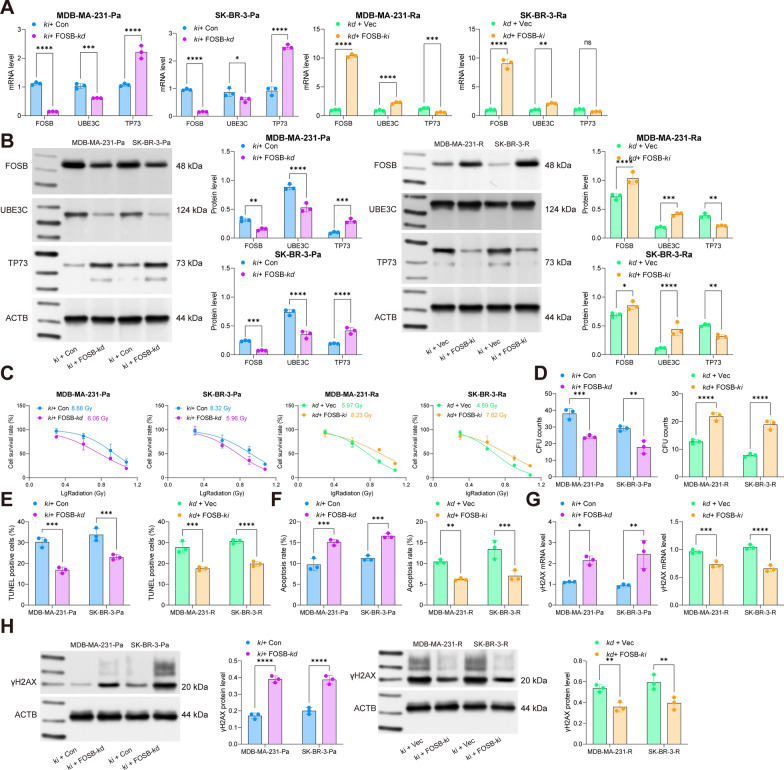
FOSB knockdown suppresses radioresistance of BRCA cells. A, RT-qPCR and WB analyses for mRNA (**A**) and protein (**B**) levels of FOSB in MDA-MB-231 and SK-BR-3 cells (R and Pa) after further sh-FOSB or oe-FOSB transfection; **C** sensitivity of MDA-MB-231 and SK-BR-3 cells (R and Pa) to different doses of irradiation ( 0, 2, 4, 8, and 12 Gy) analyzed by the CTG kit; **D** number of colonies formed by R or Pa BRCA cells under 8 Gy irradiation determined by colony formation assay; E, apoptosis of cells analyzed by flow cytometry; **F** number of apoptotic bodies formed by cells analyzed by TUNEL assay; **G**, **H**, RT-qPCR and WB assays for the mRNA (**G**) and protein (**H**) expression of γH2AX in cells. Data are expressed as the mean ± SD. Three repetitions were performed. Differences were analyzed by two-way ANOVA. **p < 0.01, ***p < 0.001

In order to identify the role of FOSB in the radiosensitivity of BRCA cells, the MDA-MB-231-R and SK-BR-3-R cells administrated with UBE3C-kd were further administrated with FOSB-ki, and the MDA-MB-231-Pa and SK-BR-3-Pa cells overexpressing UBE3C were further transfected with FOSB-kd. It was found that overexpression of FOSB in BRCA cells significantly elevated the UBE3C expression but suppressed TP73 expression. Silencing of FOSB in parental cells led to reverse trends (Fig. 7A, B). Thereafter, it was found that the radioresistance in cells blocked by UBE3C knockdown was rescued by FOSB overexpression, as manifested by reduced cell apoptosis, decreased γH2AX expression, and increased cell colonies by 8 Gy irradiation (Fig. 7C–H). On the other hand, in parental cells, the radioresistance increased by oe-UBE3C was reduced by FOSB silencing (Fig. 7C–H).

### LINC00963 binds to FOSB to activate UBE3C expression

**Fig. 8:**
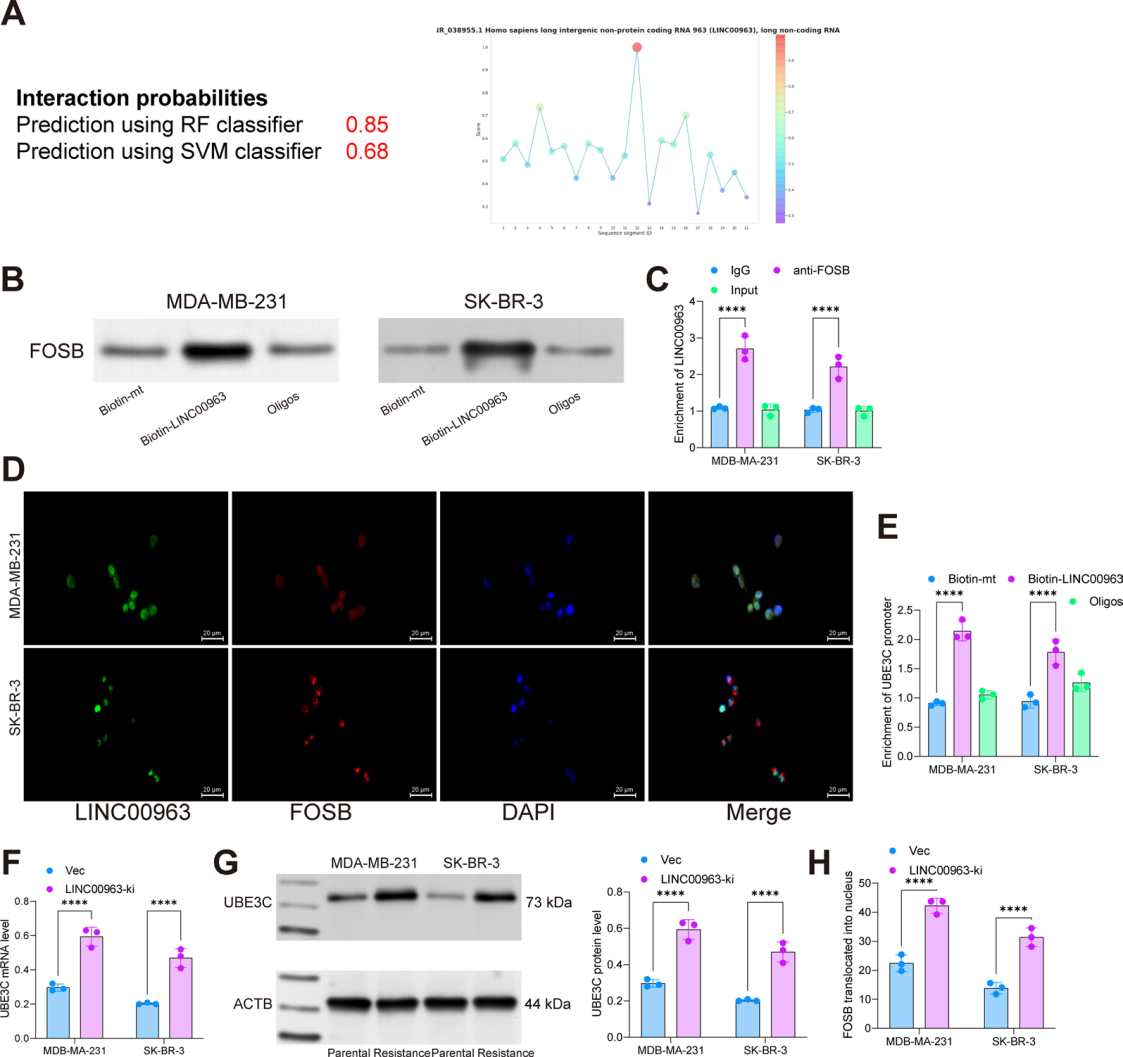
LINC00963 binds to FOSB to activate UBE3C expression. **A** RNAs binding to UBE3C and proteins binding to LINC00963 predicted by CatRapidomics analysis; **B** binding between LINC00963 and FOSB examined by biotin-LINC00963-based RNA pull-down assay; **C**, bidning between LINC00963 and FOSB validated by RIP-qPCR assay; **D** binding between LINC00963 and FOSB validated by MDA-MB-231 and SK-BR-3 cells; **E** enrichment of UBE3C promoter fragments in the complexes pulled down by biotin-LINC00963 examined by qPCR analysis; **F**, **G** RT-qPCR and WB assays for the mRNA and protein levels of UBE3C in MDA-MB-231 and SK-BR-3 cells overexpressing LINC00963; **H** nuclear translocation of FOSB in LINC00963-overexpressing MDA-MB-231 and SK-BR-3 cells determined by immunofluorescence staining. Data are expressed as the mean ± SD. Three repetitions were performed. Differences were analyzed by two-way ANOVA. **p < 0.01, ***p < 0.001

We previously reported that LINC00963 plays significant roles in the radioresistance in BRCA [24]. Moreover, transcription factors can serve as RNA binding proteins of lncRNAs to regulate the transcription of downstream genes [26, 27]. Interestingly, we obtained from the CatRapidomics system that LINC00963 could bind to FOSB protein while FOSB could bind to UBE3C promoter (Fig. 8A). To validate the binding sequence of LINC00963 with FOSB, we predicted the binding sequence of LINC00963 with FOSB in the RBPsuite website (http://www.csbio.sjtu.edu.cn/bioinf/RBPsuite/), which suggests that the 900–1000 bp and 1200–1300 bp regions of this lncRNA have the highest bindings scores with FOSB. Furthermore, we performed Biotin-labeled RNA pull-down assay in MDA-MB-231 and SK-BR-3 cells and identified an enrichment of FOSB fragments in the complexes pulled down by Biotin-LINC00963 (Fig. 8B). Moreover, the RIP-qPCR assay also showed that anti-FOSB could enrich LINC00963 (Fig. 8C). Likewise, the double-label immunofluorescence assay also showed that LINC00963 could bind to FOSB (Fig. 8D). To identify whether LINC00963 interacts with FOSB to regulate UBE3C expression, we further analyzed the UBE3C promoter fragments in the complexes pulled down by Biotin-LINC00963. As expected, the Biotin-LINC00963 enriched more UBE3C promoter fragments than Oligo or Mutant did (Fig. 8E). Moreover, overexpression of LINC00963 was found to elevate UBE3C expression in cells (Fig. 8F, G). The immunofluorescence staining showed that overexpression of LINC00963 promoted the nuclear translocation of FOSB in MDA-MB-231 and SK-BR-3 cells (Fig. 8H). Collectively, it can be inferred that LINC00963 binds to FOSB and promotes its nuclear translocation, which therefore activates UBE3C transcription and expression, leading to ubiquitination-dependent degradation of TP73 and increased radioresistance.

## Discussion

Clinically, acquired radioresistance following irradiation therapy remains a major causative factor for tumor recurrence and poor prognosis and patients [28], highlighting the need for more effective strategies to enhance radioresponse. In this study, through comprehensive bioinformatics analyses and functional experiments, we identified that the aberrant upregulation of UBE3C in BRCA cells following radiotherapy is a key contributor to radioresistance, mediated by its ubiquitination regulation on TP73. Furthermore, we found that the UBE3C upregulation is partly due to the interaction between LINC00963 and FOSB.

GEO datasets have been increasingly used as advanced and convenient tools for the quick screening of candidate genes related to specific biological processes including radioresistance in cancer [29–31]. In this study, by analyzing GSE31863 and GSE101920 datasets and querying TCGA-BRCA database, we obtained UBE3C as an upregulated gene in BRCA tissue samples following radioresistance and its elevation was linked to poor radioresponse. Indeed, we identified increased mRNA and protein levels of UBE3C in induced radioresistant BRCA cell lines compared to the parental cell lines. Previous studies have indicated the promoting roles of UBE3C in the malignant phenotype of tumor cells such as growth, proliferation, and dissemination in BRCA and gastric cancer [13, 32], which were reportedly attributive to the activation of the oncogenic β-catenin. In a recent work by Xu et al. UBE3C has been reported to accelerate proliferation, migration, invasion, angiogenesis, and resistance to death of clear-cell renal-cell carcinoma cells through by inducing ubiquitination of phosphatidylethanolamine binding protein 1 [33]. The oncogenic property of UBE3C is thus nothing new, but its correlation with radioresistance remains elusive and intriguing. By inducing UBE3C knockdown in two radioresistant BRCA cell lines while UBE3C knock-in in two parental BRCA cell lines, we observed that the UBE3C was linked to proliferation, resistance to death and DNA damage, and tumorigenic activity of cells under irradiation exposure. Therefore, we confirmed that the UBE3C functions as a decisive factor leading to increased radioresistance.

Thereafter, by performing Co-IP, we identified TP73 as a highly reliable target of UBE3C among the candidates predicted from the Ubibrowser system. Unlike TP53, TP73 has no mutation reported [18], but the full-length TP73, mainly TAp73 reportedly mimics p53 function including the enhancement of sensitivity to radiotherapy in experimental systems [34, 35]. Indeed, it has been reported as a credible biomarker predictive of cancer regression and favorable prognosis of patients [36]. Under normal physiological circumstances, the TP73 protein levels are in general quite low, but TAp73 accumulates and stabilizes in response to irritation [19]. However, we found that the UBE3C upregulation following radiotherapy induced ubiquitination and degradation of the TP73 protein. The p53-like anti-proliferative and pro-apoptotic properties of TP73 make it as an important determinant of resistance to not only chemotherapy but also radiotherapy [35, 37]. However, the direct influence of TP73 on chemoresistance in human cancers remains largely unknown. Here in this work, the fact that TP73 knockdown suppressed whereas its upregulation increased the sensitivity of BRCA cells to irradiation evidenced its supportive role in radiosensitivity.

By querying the CatRapidomics system, we obtained that LINC00963 could bind to FOSB protein while FOSB could bind to UBE3C promoter. We previously found that LINC00963 promoted radioresistance in BRCA by sequestering microRNA-324-3p and inducing ACK1 overexpression [24]. The interaction with other RNAs represents one of the classic functional mechanisms of lncRNAs. In addition, they can bind to DNA or transcription factors directly to modulate gene expression in the transcription level [38]. Upregulation of FOSB has been detected in highly metastatic triple-negative BRCA [39]. Likewise, FOSB has been identified as one of the key transcription factors aberrantly expressed in BRCA patients. Although there has been no exact evidence concerning the function of FOSB in radioresistance, a previous work by Bandey et al. demonstrated that high expression of FOSB in tumor tissues was linked to elevated granulin precursor that was correlated with the expression of genes related to DNA repair [40]. Here, we validated that the binding between LINC00963 and FOSB promoted the nuclear translocation of FOSB, which led to increased transcription of UBE3C. Indeed, artificial downregulation of FOSB was found to reduce radioresistance in two BRCA cell lines.

Despite the inspiring findings, there remains several limitations of the study. First, we did not introduce mutated segments of LINC00963, making the exact region of sequence correlated with its specific biological activity still elusive. Meanwhile, in terms of the ubiquitination assays, performing UB pulldown and mass spectrometry analysis would better present the ubiquitination enrichment. These experiments were not included in this work primarily due to the funding and time limitations. Furthermore, the LINC00963 to TP73 axis represents one of the possible mechanisms; however, according to the bioinformatics analyses, there might be more regulatory axis involved in the regulation of radioresistance in BRCA. We would focus on these issues in our future studies.

## Conclusion

In conclusion, the present work demonstrates that LINC00963 binds to FOSB to increase its nuclear translocation and the subsequent transcription activation of UBE3C, which induces ubiquitination and protein degradation of the tumor suppressor TP73 (graphical abstract). This study provides evidence that UBE3C and TP73 have specific roles in radioresponse and might shed new lights in the management of radioresistant BRCA. Targeting any member of the LINC00963/FOSB/UBE3C axis, or specific restoring TP73 may help enhance radiosensitivity of BRCA cells. Furthermore, we would like to identify more molecules, and fix the current gaps of the present work in our ongoing research.

## Data Availability

All data generated or analysed during this study are included in this article. Additional raw data may be available from the corresponding author for reasonable reasons.
